# Multi-Sensor-Fusion Approach for a Data-Science-Oriented Preventive Health Management System: Concept and Development of a Decentralized Data Collection Approach for Heterogeneous Data Sources

**DOI:** 10.1155/2019/9864246

**Published:** 2019-10-08

**Authors:** Sebastian Neubert, André Geißler, Thomas Roddelkopf, Regina Stoll, Karl-Heinz Sandmann, Julius Neumann, Kerstin Thurow

**Affiliations:** ^1^Institute of Automation, University of Rostock, Rostock 18119, Germany; ^2^Center for Life Science Automation (celisca), University of Rostock, Rostock 18119, Germany; ^3^Institute for Preventive Medicine, University of Rostock, Rostock 18119, Germany; ^4^S&N Datentechnik, Rostock 18055, Germany

## Abstract

Investigations in preventive and occupational medicine are often based on the acquisition of data in the customer's daily routine. This requires convenient measurement solutions including physiological, psychological, physical, and sometimes emotional parameters. In this paper, the introduction of a decentralized multi-sensor-fusion approach for a preventive health-management system is described. The aim is the provision of a flexible mobile data-collection platform, which can be used in many different health-care related applications. Different heterogeneous data sources can be integrated and measured data are prepared and transferred to a superordinated data-science-oriented cloud-solution. The presented novel approach focuses on the integration and fusion of different mobile data sources on a mobile data collection system (mDCS). This includes directly coupled wireless sensor devices, indirectly coupled devices offering the datasets via vendor-specific cloud solutions (as e.g., *Fitbit*, San Francisco, USA and *Nokia*, Espoo, Finland) and questionnaires to acquire subjective and objective parameters. The mDCS functions as a user-specific interface adapter and data concentrator decentralized from a data-science-oriented processing cloud. A low-level data fusion in the mDCS includes the synchronization of the data sources, the individual selection of required data sets and the execution of pre-processing procedures. Thus, the mDCS increases the availability of the processing cloud and in consequence also of the higher level data-fusion procedures. The developed system can be easily adapted to changing health-care applications by using different sensor combinations. The complex processing for data analysis can be supported and intervention measures can be provided.

## 1. Introduction

### 1.1. Project: p^2^Health

For the application of preventive medicine measures (including occupational health and social medicine) mostly the individual acquisition of physical, physiological, and psychological data are required beforehand. Therefore, comprehensive telemonitoring systems are required, which allow the measuring of customer-specific data in the usual daily routine (e.g., at work, in leisure time, at sports, during the night). In order to get real and unaltered feedback, the minimization of measurement influences on the customers is a very important factor. The choice of required parameters strongly depends on the investigation focus. Consequently, a wide range of mobile and preferable lightweight and small sensor solutions is required to cover different medical questions.

**Figure 1:**
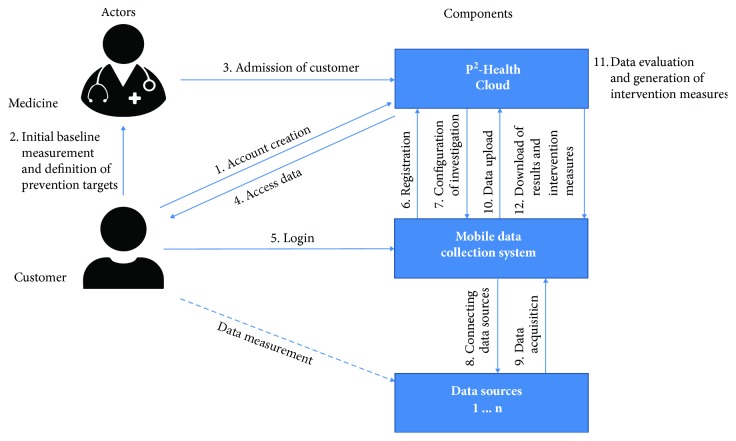
Fundamental concept of the project *p^2^Health*.

Within the project *p^2^Health* an extended concept for a personal preventive health management (*p^2^Health*) is to be implemented. It combines a personal mobile data monitoring by wearable sensors with a preventive medical support. This support includes, among others, an initial medical checkup, intelligent data interpretation, and provision of user-specific intervention measures by a processing cloud-solution. The approach focuses on offering evidence-based and medically assisted possibilities for customers in all living environments to monitor their activities, metabolic states, and mental health under consideration of environmental parameters.  Figure 1 shows the general concept of the project.

Accordingly, a solution is required, which integrates the different types of sensor systems and prepares the inhomogeneous data for processing and interpretation in the cloud.

### 1.2. State of the Art

The *quantified self* (QS) movement [1, 2] led to the introduction of numerous wearable, primary wrist-worn, sensor solutions to the market, which are made for the application in daily routine (compact and comfortable). The measured parameters include e.g., step count, traveled distance, caloric analysis, energy and oxygen consumption, fitness tracking, heart rate and sleep stages, and additional features, as e.g., food documentation, data sharing, and alarm functions [3, 4]. These systems usually do not achieve the quality of conventional methods in laboratories [5–7]. In addition, they partially have strong derivations if the application range differs from the usual one. In [8, 9], for example, the derivations of step counters for different movement speeds are presented. But there are also strong differences in quality between the offering device manufacturers as exemplarily shown for energy expenditure measurements in [10]. Nevertheless, they offer a sufficient compromise between data quality and usability/comfort [11] for different medical investigations in field [12–15].

Leading providers for such sensor solutions are e.g., *Fitbit* [16], *iHealthLabs* [17], or *Nokia* [18]. They channel the data of their sensor units via their own smart-device applications to their cloud servers [19]. These cloud servers offer comprehensive interfaces, which allow specific data requests. Some current developments of telemedical monitoring systems replace the common integration of single Bluetooth-devices by the integration of cloud-solutions. This avoids the development of specific smart-device applications as well as the integration of sensor systems. Several developments for different applications can be found in the literature, which use these sensor systems and solely integrated the respective cloud servers for the health-data access. *iCardia* [20] is a platform for supporting people in cardiac rehabilitation by tracking their activity. These data are acquired by wrist-worn wearable devices from *Fitbit* and are stored in the respective provider-cloud server. The *iCardia*-cloud queries current data sets (notified by *Fitbit's* Subscription API) from the *Fitbit* cloud and provides these data in the iCardia clinical app (dashboard). Here the data are analyzed by specialists in cardiac rehabilitation, which send personalized feedback via SMS to the participants. The platform *MyHealthAvatar* (MHA) [21] collects life-logging data, including health and social media data, to provide user-specific information for interdisciplinary healthcare research and collaboration. The MHA server acts as a central data hub, which aggregates different data from mobile apps, wearable devices, and social media (e.g., *Twitter*, *Facebook*, *Nike+ Fuelband*, *Jawbone Up*, *Google MyTracks*). In more recent studies, MHA contributes this functionality to the iManageCancer project, which supports prostate and breast-cancer patients by e.g., tailored information provision and a personalized risk assessment [22]. *MoC* (Mobile Cloud) *Medicare* [23] is a mobile platform, which is developed to track the health status (as heart rate, sleep time, and burnt calories) and to detect fall situations of elder people by wrist-worn sensors to obtain a higher degree of safety. In contrast to the previous platforms, where the respective cloud server solutions are connected, every instance of *MoC Medicare* mobile application establishes an individual connection to the provider cloud server to acquire the user's health data and to forward them to the central data storage (*Amazon* cloud server). In addition, the *MoC Medicare* application has an implemented fall-detection algorithm, which uses the acceleration data of the mobile device for pattern recognition. In case of detecting a fall, the app is sending a notification to a registered emergency contact.

Mobile health toolkits [24] as e.g., *Apple HealthKit*, *Google Fit* or *Samsung Health* offer cloud-based services, which function as repository for health- and fitness- related data. Besides vendor-specific sensor-devices and app solutions, also selected third-party products can be integrated and merging of data from different sources is supported. The architecture of these systems for the integration of third-party wearable devices can be divided into two general approaches. The first one offers the integration of the corresponding third party apps into the toolkit app on the mobile device and the subsequent upload to the provider cloud [24]. This, for example, is applied by *Apple HealthKit* and *Samsung Health*, whereby these solutions allow only less third-party integrations, compared to *Google Fit*. The approach applied by *Google Fit* offers the up- and download of data [25] to/from the *Google Fit* cloud for third-party clouds and apps. In this case, the third-party apps do not interact with the *Google Fit* app on the mobile device [24]. All of these health-toolkit solutions permit the up- and download of data by APIs (application programming interfaces) and they also provide an upload opportunity of additional data into their clouds, independent of the data source. Due to the shutdown of the *Google Fit* websites in March 2019, a trend shift to access data predominantly via mobile-device apps can be assumed.

Health toolkits are primarily used in fitness- and activity-related studies, where the access to data already stored in the clouds for statistical analysis is required (as e.g., in [26, 27, 28]). Therefore, the data mostly are downloaded or requested on a server level. Only a few research solutions feature additional integrations in health toolkits on mobile devices. In [29], for example, physical activity data and heart rate from *Google Fit* and* Apple HealthKit* are used to expand the seventh population based *Tromsø Study* (Tromsø 7, in Tromsø, Norway), which is repeated every 5–8 years. Reason of these investigations is the high prevalence in cardiovascular diseases in Norway. Via the *Tromsø Study* application on the mobile device, the participants' data are once downloaded from *Google Fit* or *Apple HealthKit* Cloud (security clearance by participants) and sent to a web-service in the *Tromsø Study* backend. The web-service realizes the processing/adaption of data into the required format to store it in EUTRO, the main research data storage in the *Tromsø Study*. A further study uses *Google Fit* to store fitness data under consideration of saving energy as described in [30]. Here the *Google Fit*-compatible Sony Smartband 2 (Minato, Tokio, Japan) was used to track patients with heart diseases. Accordingly, the data are stored in *Google Fit* and are queried by an Android app, which detects situations with an increased risk of heart attacks for a selected day. The detection is based on thresholds, calculated by a weighted average of the heart rate.

However, the common integration of directly coupled wearable sensors (by e.g., Bluetooth, ANT, Zigbee or WiFi) with disclosed communication protocols or provided software development kits (SDKs) is still essential, especially if real-time conditions or less common, respectively, special parameter sets are required. Moreover, these sensor solutions have a higher variety regarding the available parameters [31, 32] and vendors (depending on the requested parameters and sensor specifications) [33, 34]. In addition, they allow more complex installation arrangements, as for example, the measurement of respiration or body orientation by chest belts [35–38] or motion patterns by distributed body-wide sensors [4]. The typical architecture for directly coupled wearable sensors includes one or more sensor solutions, which are connected to a mobile application. These applications often realize the processing and presentation of data and also allow forwarding the data to a web server or cloud. Several applications using such sensor solutions are summarized in [39]. Additionally, the parallel acquisition of environmental parameters with wearable sensors plays an increasingly significant role [40–45] in preventive medicine. Consequently, the flexible combination of different wearable data sources, depending on the individual investigation focuses and under consideration of wearing comfort and adequate data quality, is a current engineering challenge in this field.

The introduced examples for both sensor-integration approaches show that in current developments usually only one approach is used. Accordingly, in the literature only one development could be found, which deals with the fusion of directly coupled wearable sensors and vendor-specific cloud servers (here collectively designated as heterogeneous data sources). This solution pursues an approach for patients with hypertension, where heterogeneous data sources for blood pressure, weight, and activity tracking are combined [44]. The data fusion and the control of the process workflow in this solution are centrally realized by a BPM (business process management)-supported server/cloud platform. It collects data from vendor-clouds and smart devices via RESTful services (REST—Representational State Transfer) and also integrates web-connected remote sensors for ambient monitoring (temperature and atmospheric pressure). Depending on the collected data and individual preferences, the system offers recommendations to the patients for healthy habits and notifies them, if any risk factors are detected. The decision making for the recommendations is an integrated part of the BPM-model.

As is apparent from the previous considerations, the developments mostly concentrate on directly or indirectly coupled wearable sensors only. For preventive-medicine purposes both approaches are important regarding the measured parameters, the respective advantages (e.g., data quality, flexibility, and usability) and their availability on the market. The fusion of data for such wearable sensors would cover a wide range of scenarios in preventive medicine. Accordingly, in this work the conception and development of an appropriate solution approach for a mobile data collection system (mDCS) [45] will be proposed.

In the materials and methods section, at first the system requirements and development issues are outlined.  Section 2.2 presents the system structure and concept in relation to the outlined development tasks. In Section 2.3, the communication between the involved system components and their interactions is described. In the results and discussion section, the smart-device application *MobMedApp* is presented, including the software architecture, the connection management, and the sensor data handling. *To prove the functionality* of the concept an example application is offered in Section 3 before the paper is summarized in Section 4.

## 2. Materials and Methods

### 2.1. System Requirements and Development Issues

Due to the high variety of different key issues and the need of more precise and realistic data sets in preventive medicine, the use of mobile telemonitoring solutions becomes very important. From a medical perspective the following requirements for these solutions arise:

- light-weight and easy to wear (e.g., wireless solutions) system components,
- simple and intuitive system handling (usability),
- long service life,
- high data security and integrity,
- automatic data processing and fast data availability (if necessary),
- high adaptability for different parameters (by sensors and questionnaires), data sources and processing algorithms.

Especially, the last point has higher priority in preventive medicine than in most other medical fields using mobile telemonitoring solutions due to the wider application range. In addition to the medical demands, the following technical aspects have to be considered:

- flexible integration and adaption of diverse data sources to permit individual application scenarios,
- fast provision of processing results for less complex cloud-solution infrastructures, especially if larger user groups measure in parallel (availability of processing components),
- clearly allocated functionalities for the system components and clearly defined interfaces to allow a flexible adaption or exchange of single components,
- consideration of extension possibilities for a simple upscaling of the system for higher number of users (scalability).

The medical and technical requirements demand a holistic consideration of the system's development. Thus, the following, closely related scientifically relevant development issues do arise:

- *Data source integration*—Preventive medicine investigations increasingly demand adaptive integration solutions of the abovementioned heterogeneous data sources to allow the adaption to the wide range of different investigation settings. Additionally, the acquisition of subjective user information is still a proven approach, especially if psychological-related investigations (as e.g., mental stress studies) are targeted.
- *System structure conception*—Many telemonitoring systems (especially in research or for startup companies) are limited to small fields of application. They are not based on complex computer structures and consequently require optimization strategies regarding the system design (e.g., modularization, load distribution, optimized system utilization) to ensure adequate system availability and flexibility.
- *Sensor data fusion*—Final aim of the system development is an adaptive complementary as well as cooperative data fusion. Different sensors work together to obtain comprehensive information about an investigation state and to derive indirect measurable parameters that could not be achieved by one sensor only [11, 46, 47]. Therefore, the mDCS has to provide a low-level fusion [48, 49], which is the base for higher level fusion techniques (feature and decision fusion [11, 48, 49]) and for the intelligent data interpretation taken over by superordinated system components in a hierarchical structure [50].

The novelty of the described solution approach is the decentralized sensor integration and low-level fusion (related to the processing cloud-solution) of all mobile, heterogeneous data sources on user-related nodes. Thus, a more stringent structural decoupling of the systems' layers and a better utilization of resources can be achieved. Most of the other current developments focus on one kind of data source only or on a central fusion of the measurement data.

### 2.2. System Structure and Concept

The components of the preventive health management system are hierarchically structured in accordance to the three typically used levels of mobile telemonitoring systems.

- Data processing level: *p^2^Health cloud*.
- Data collection level: mobile data collection system with *MobMedApp*.
- Data acquisition level: data sources as mobile sensor solutions/smart watches.

**Figure 2:**
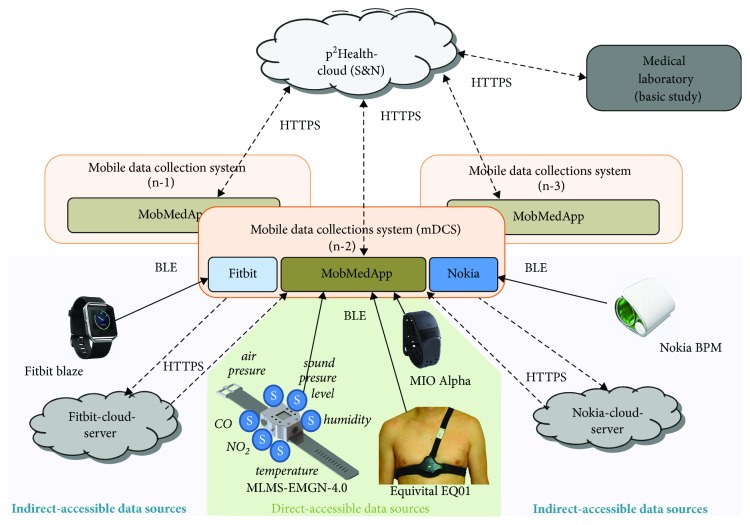
General system structure with focus on the mDCS.

The highest level of the developed preventive health-management system's structure is represented by the *p^2^Health cloud* (based on Microsoft Azure cloud computing platform). It provides the user's access (including medics and customers) and combines results from baseline examinations made in medical laboratories with investigations in field done by the mDCS (see Figure 2). The baseline examinations include, for example, the determination of body composition and metabolic rate, a (spiro-)ergometry and a psychological load analysis (e.g., by VTS—Vienna Test System), which deliver individual initial parameters for the data analysis. The cloud obtains the data from the laboratory, the mDCS, and official reference databases. It also executes data-analysis routines (including feature and decision fusion) resulting in individual interventions' measures for the customers. The data analysis is primarily based on a selection of neural-network/fuzzy models [51], which work problem-oriented and thus have to be updated or complemented for new issues. The *p^2^Health cloud* offers specific web portals for users and medics. These web portals obtain an individual investigation plan for every user and include the acquired data as well as the processing results. While the users primarily use the system to inform themselves and to export data, the medics also use the portal to create and edit investigation plans including the required parameters, the processing and general configurations.

The mDCS are represented by smartphones, which have sufficient interface options for the connection to the data sources and the *p^2^Health-Cloud*. Basis of the mDCS is the smart-device application *MobMedApp*, which is responsible for the establishment and maintenance of the connections to all required system components. The collection and preparation of individually required data and the presentation of partial outcomes are additional tasks of the *MobMedApp*. It manages the data flow for the individual customer's demands and transfers the data for the further processing to the *p^2^Health-Cloud*. For the collection of measurement data in the *MobMedApp,* the following sources for the acquisition of data are distinguished:

- *Directly accessible data sources* [52]—wireless sensor devices (equipped with e.g., *Bluetooth low energy* (BLE), *Bluetooth Classic* or *Ant+*) with open communication protocol or provided SDKs by the vendors as e.g., *Mio™ Alpha* (*Mio™ Global*), *Equivital™* (*Hidalgo Ltd.*) or the self-developed environmental monitoring system MLMS-EMGN-4.0 [53, 54].
- *Indirectly accessible data sources* [52]—provider-cloud servers, as e.g., by *Fitbit* (San Francisco, CA; USA) or *Nokia* (Espoo, Finland), offer data of their provider-specific sensor units (usually fitness watches); no open wireless communication to the sensor devices supported.
- *Subjective and objective data entry*—query options (e.g., questionnaires and chronometrage) integrated in *MobMedApp* for the customers' subjective and objective assessment.

Generally, indirectly accessible data sources are structurally more complex than direct ones due to the high amount of involved system components (e.g., provider app, provider-cloud servers and potentially included processing services). They are also less flexible regarding the configuration, since many options are prescribed by the providers. Benefits from this approach are e.g., an independent data buffering by the provider-cloud servers, an often included data processing and a significant reduction of the integration effort for a wide range of different sensor units, dependent on the provider's product range. Consequently, new or extended parameters only require the adaption of the interface.

**Figure 3:**
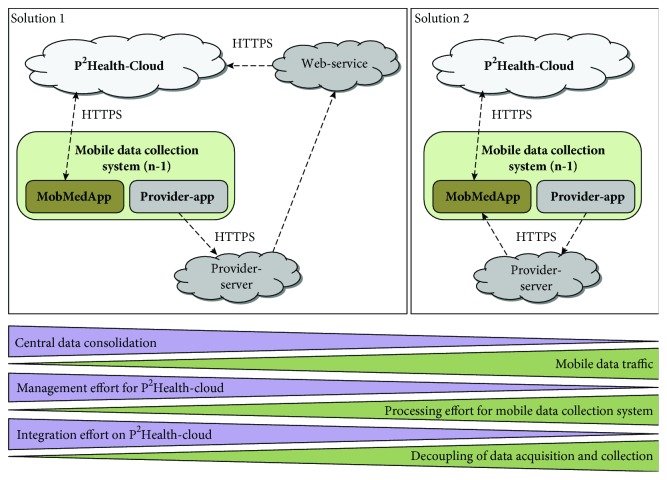
Comparison of two solutions for the integration of provider-cloud servers for a process-specific data request.

In Figure 3, two solutions for the integration of indirectly accessible data sources are presented and compared regarding important divergent properties. In solution 1, a web-service realizes the connection between the provider-cloud server and *p^2^Health-Cloud*. In solution 2, the data are routed via the mDCS to the *p^2^Health-Cloud*. Both solutions can be used to perform appropriate procedures for data consolidation and pre-processing.

Solution 1 uses a service-oriented architecture (SOA) [55] for the implementation of web-services, which can be a separated solution or being combined with the *p^2^Health-Cloud* structure (as comparatively in [56]). The *p^2^Health-Cloud* initiates data transfers via specific web-services to the provider-cloud servers if data are required for the data-analysis routines. This solution requires the *p^2^Health-Cloud* to partially realize pre-processing procedures as well as the final synchronization of data delivered by the *MobMedApp* and from other web-services, which connect further provider-cloud servers. In this case the* p^2^Health-Cloud* has a high integration effort and requires a comprehensive management regarding the periodical and parallel connections to the specific data-source accounts. Moreover, the cloud has to handle all accesses including the consideration of restricted access rights (pre-determined by the providers) and the individual customer's registration.

In contrast to that, solution 2 encapsulates the sensor fusion completely on the mDCS and relieves the *p^2^Health-Cloud* regarding the handling of several sensor-data-related connections. The cloud, in this solution, is focused on the data analysis and only handles the connection to the mDCS and medical laboratories. The *MobMedApp* samples and consolidates user-specific data from the different sources, manages the data handling, and delivers them to the *p^2^Health-Cloud* via an appropriate interface protocol. All management tasks and pre-processing procedures are decoupled from the *p^2^Health-Cloud* and remain on the mDCS. Thus, the mDCS represent decentralized, customer-specific connection adapters and data concentrators. Disadvantages of this solution are the increasing mobile data traffic and the higher processing effort on the individual mDCS. These can be reduced by balanced polling frequencies dependent on investigation focuses. However, solution 2 is preferred due to the more stringent hierarchically structured approach, the instant availability of all primary data on the mDCS and the appropriate clearly divided task distribution, which also supports the system's scalability.

The connection to health toolkits (as introduced in Section 1.2) instead of integrating several vendor clouds is a suitable alternative to reduce the integration effort of indirectly accessible data sources. However, in the presented development a strong focus is on Fitbit-devices, which require additionally third-party adapters to transfer data into the *Google Fit* cloud (available for Android and Fitbit in combination). Consequently, this increases the structural complexity and the communication effort on the mobile device. Furthermore, while the users do not necessarily use Google Fit, the use of the vendor-specific clouds is of course binding. In addition, also the directly accessible data sources and questionnaires as well as the data preprocessing have to be considered in the development. Thus, initially a single data node (mDCS) without health toolkits is preferred.

### 2.3. Communication

In the health-management system's structure, three different types of connections, originating from the mDCS, are distinguished: the connection to the *p^2^Health-Cloud*, to the provider-cloud-servers, and to the directly accessible wireless devices (currently considered standards: BLE and *Bluetooth Classic*). The *p^2^Health-Cloud* and the provider-cloud-servers follow the same technical concept. The complexity of the protocol and the data volume are higher with the connection to the *p^2^Health-Cloud*.

For connections to the Bluetooth devices, the generic attribute (GATT) profile (for BLE) and the still often used serial port profile (for Bluetooth Classic) are supported. The *Bluetooth*-device integration follows the standard procedure and will thus not be described here.

Separated connections via appropriated services are used for the API-dependent data exchange with the *p^2^Health-Cloud* and the provider-cloud-servers. The latter require the creation of a personal and unique developer account for each client and provider, parallel to the usual user account. Both accounts have a one-to-one relationship. In the authorization process a token is generated, which allows access to the customer's data (*Fitbit*: OAuth 2.0, time-limited tokens of one or eight hours, refreshing tokens available; *Nokia*: OAuth 2.0, no time limits for tokens). HTTPS is used for the data transfer from the provider-cloud-servers; the data are anonymized. For the communication between the *p^2^Health-Cloud *and the mDCS also the OAuth 2.0 authorization as well as HTTPS and anonymized data are used.

**Figure 4:**
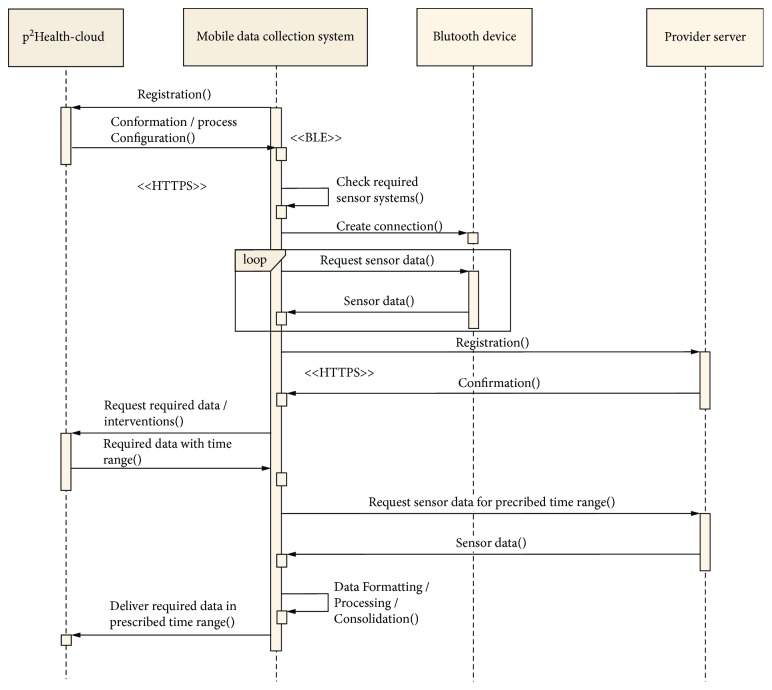
Simplified sequence diagram with the initialization procedure of the connections (between the mDCS, the *p^2^Health-Cloud,* and the sensor solutions) and the control of the sensor data traffic.

In Figure 4 a simplified sequence diagram, comprising the initialization of the connections and the general control of the data traffic by the mDCS is shown. The *MobMedApp* realizes the communication management for the individual customer's process on the mDCS. The first step is the establishment of a connection to the *p^2^Health-Cloud *to receive all necessary customer-specific configurations of the monitoring process. Dependent on the required data, the *MobMedApp* establishes the connection to available sensor solutions. The used Bluetooth devices require a periodical data request and a temporary data storage. Data from the provider-cloud servers only need to be requested if the data transfer to the *p^2^Health-Cloud* is imminent.

## 3. Results and Discussion

### 3.1. System Implementation with *MobMedApp*

#### 3.1.1. Architecture of *MobMedApp*

**Figure 5:**
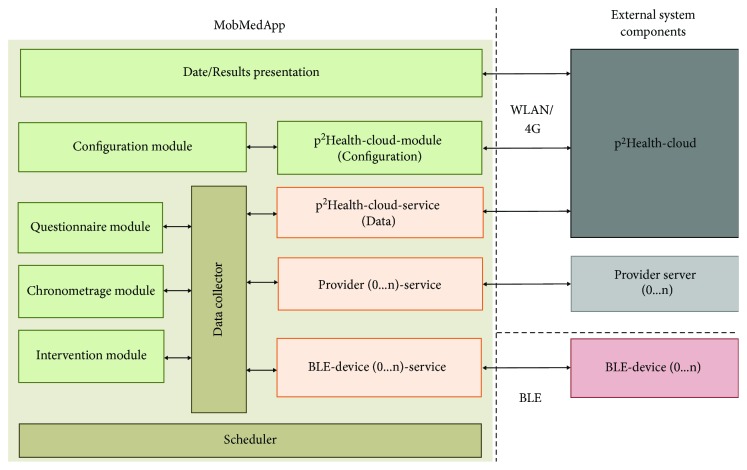
Architecture of the *MobMedApp*.

The *MobMedApp* is the central data node for the customer's individual monitoring. Therefore, different communication channels, the data management, and the processing, as well as support of the mobile customer interfaces are combined. In Figure 5, the used architecture of the *MobMedApp* including potentially connected external system components is shown.

For every communication channel, a service instance is generated to maintain the connection to the external system component independently of the *MobMedApp*-status (e.g., if the app is in the background of the operating system) and to adapt the device-specific protocol. The incoming data are sampled by the data collector, which realizes data consolidation, pre-processing and adaption of the required formats. It also handles investigation-related subjective and objective data inputs via questionnaires and the chronometrage-module (optional manually record of classified activities as e.g., sport, sleep, work). Data of the chronometrage allow a better temporal and load-related differentiation of the acquired measurement data and support the data analysis at the* p^2^Health-Cloud*, especially during teach and validation phases. Moreover, the data collector manages autonomously the intervention measures to be executed dependent on the *p^2^Health-Clouds* instructions.

**Figure 6:**
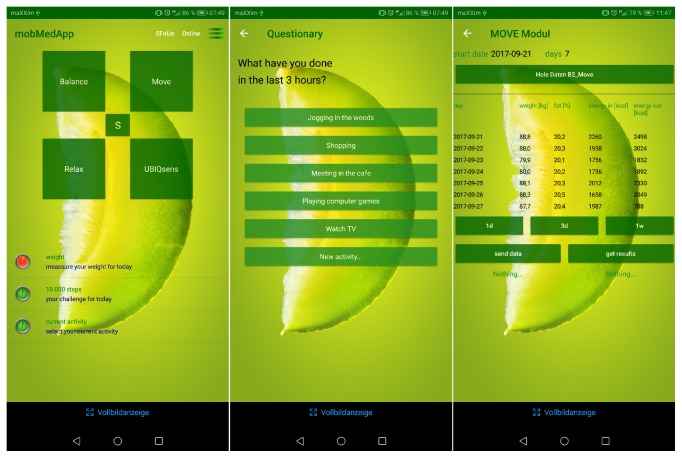
Graphical user interface (GUI) of the *MobMedApp*; left: main screen with supported investigation categories and listed intervention tasks; middle: questionnaire for individual acquisition of objective/subjective information; right: conclusion of acquired data for current investigation focuses.

A task scheduler manages the user event queue according to the user-related configurations by the *p^2^Health-Cloud*. These configurations are transferred into a task table, which includes the sensor data to be acquired, their units and resolutions and the upload interval to the *p^2^Health-Cloud*. Every 10 minutes the *MobMedApp *checks for changes in the task table. The task scheduler creates (or deletes) own tasks from the task table for execution in the *MobMedApp* and thus realizes the process management and dataflow control. This management includes among others reminders for the user to follow the proposed intervention measures, to measure additional parameters (as e.g., weight or blood pressure) or to fill out required questionnaires. The event queue is shown as a list with planned and outstanding tasks, ordered, and indicated by their priority (pre-announced, execution time, exceeded deadline; see Figure 6). The customers finally decide about the execution or rejection (as e.g., during the sleeping phase) of outstanding actions.

A separated module allows the presentation of processed and partially visualized results of the *p^2^Health-Cloud's* data analysis.

#### 3.1.2. Connection and Access Management

**Figure 7:**
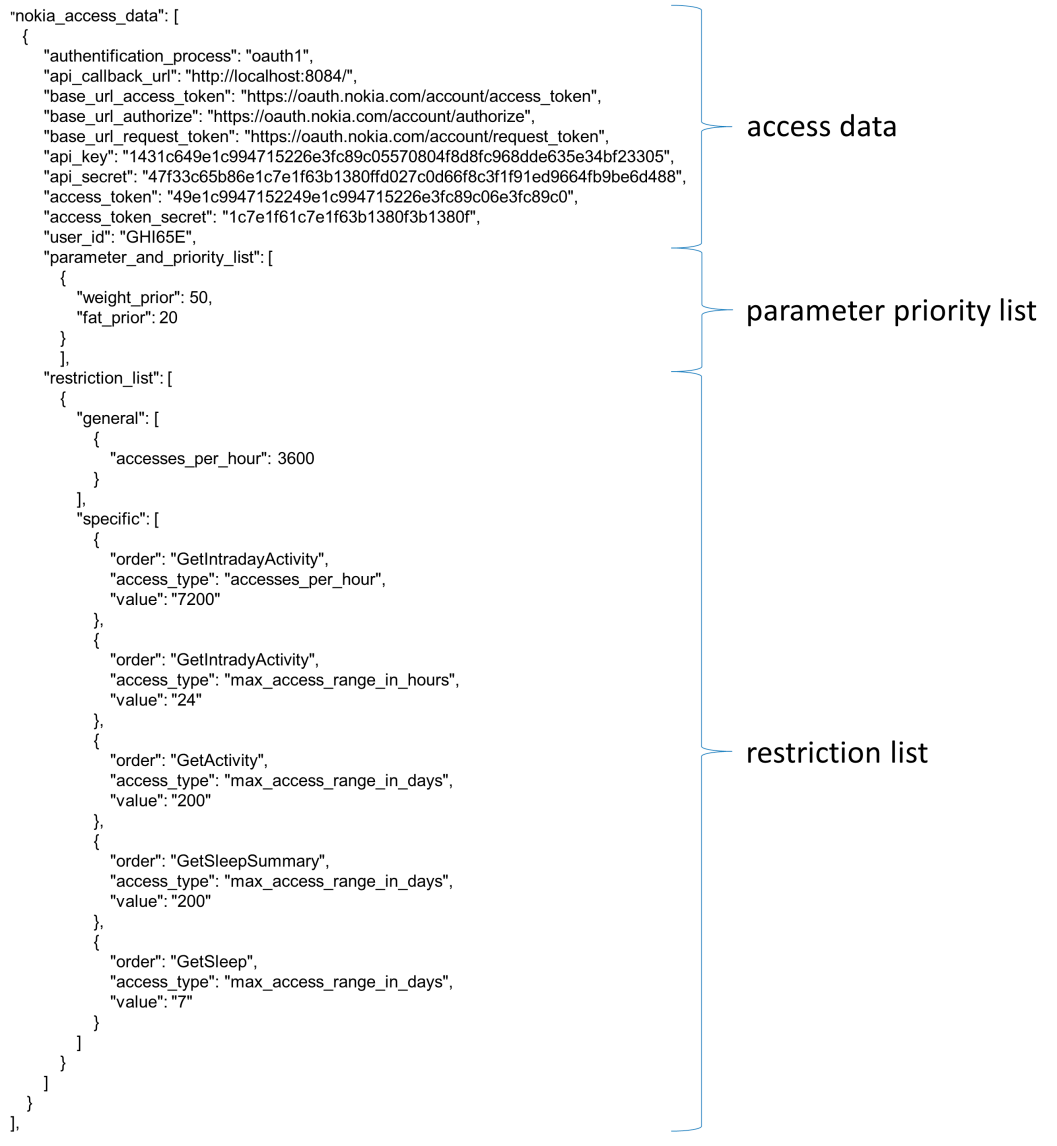
Entry of a provider-cloud server in the data-source table.

Regarding the required sensor connections the *MobMedApp* makes a choice depending on the parameters demanded by the *p^2^Health-Cloud*, the connections authorized by the users and the available sensor units. Therefore, a data-source table is used (see Figure 7), which includes the offered parameters for each sensor, the sensor's configuration sets, the access conditions, and the sensor's parameter priority. Currently, the data-source table is provided individually for every user by the *p^2^Health-Cloud*. Accordingly, only the potentially possible sensor devices and cloud accesses are considered. The data-source table is always updated after the start of the *MobMedApp *and in case of longer use additionally once per day.

The sensor-configuration sets are the base for the connection establishment to the Bluetooth sensors (e.g., available UUIDs of sensor characteristics, device names, MAC-addresses and authentication procedures) and to the sensor-provider-cloud servers (e.g., IP-addresses, user-IDs and keys, authentication procedures, refresh and access tokens of provider and query functions). The access conditions primarily focus on the data collection via the sensor-provider-cloud servers. Here restrictions regarding the number of queries per time unit and the maximum time range for data requests (e.g., time range per request of maximal 200 days) are defined. Some providers do not offer the possibility to request all available user parameters in one query. Depending on the required number of parameters and the required polling frequency, the respective restrictions can be reached and affect the data transfer. Since the synchronization between the sensor device and the provider's smartphone application and further to the provider's server is not continuous, a close to real-time data transfer is not feasible for this approach. The sensor's parameter priority is used if several sensors acquire the same parameter with a different data quality. In this case, the *MobMedApp* decides which of the acquired parameters are to be preferred for the further processing depending on the higher priority. The priority is determined by the medical group, which decides according to initial device test and application experiences.

Due to the number of accounts arising in the *MobMedApp*, and the requirements regarding a simple and safe handling of these accounts, two approaches are considered in more detail.

The first approach keeps all account information only on the mDCS. In this case, the users handle the account data in the *MobMedApp*, including the registration of user and developer accounts for each provider. This preserves the users' right of informational self-determination. In the second approach, only the *p^2^Health-Cloud*-account data are handled in the *MobMedApp*. All other access data for the user and developer accounts of the provider-cloud servers are managed centrally and can be requested via the *p^2^Health-Cloud* if required. This approach is especially convenient for technically inexperienced customers, since there is no need to register accounts or to apply account data, which significantly increases the usability. In this paper, the second approach is preferred. Nevertheless, the first approach is also considered as alternative to keep the independencies of the mDCS regarding the sensor-data-resource accesses.

#### 3.1.3. Sensor Data Management

The sensor data management in the *MobMedApp* prepares the data for the individual data-analysis methods in the *p^2^Health-Cloud*. Therefore, the cloud configures the *MobMedApp* regarding the required parameters and the necessary investigation configurations as e.g., frequency of data provision and the conditions of reminders and requests for the customer to measure data or to fill in questionnaires. Depending on these requirements the *MobMedApp* collects the data and performs the following essential tasks:

- data consolidation and synchronization,
- pre-processing,
- data selection,
- reformatting of data sets.

With the consolidation and synchronization, the requested data from available data sources are combined for the prescribed time range. The data time for directly and indirectly accessible data sources often complies with the time of the mDCS. Some sensor units with internal timing devices only have a manual opportunity for the synchronization. In this case, the approximate time offset is determined before the monitoring and considered for the subsequently received data sets.

The data management also covers pre-processing procedures, which allow the conversion of expected default units, data compression, and the generation of secondary data. The available processing procedures are predefined in the *MobMedApp* and need to be requested by the *p^2^Health-Cloud* via the respective resulting parameter. A necessary condition for the processing is the availability of the corresponding primary data. The decisions regarding the required pre-processing steps in the *MobMedApp* are made by medics and data-processing engineers. While medics use these pre-processing steps for an optimized overview of the data, the process engineers primarily use them to standardize the input conditions for their processing modules on the *p^2^Health-Cloud* for the different data sources. Among others, the following processing routines are currently included in the *MobMedApp*:

- separation of heart rate periods depending on activities (e.g., running, biking, walking) and heart-rate zones (peak, cardio, fat burn) either by day or by training session
  1. calculation of average heart rate for respective periods (per day or training session)
  2. calculation of total duration of the respective periods (per day or training session)
- calculation of average heart rate for day and night phase
- calculation of energy consumption of the different activities per day
- counting of active phases for the separated activities (e.g., walk, hike, workout) per day
- conversion to unified designations as e.g., for body orientation (upright, side, supine etc.) or sleep phases (wake, rem, light, deep)
- conversion of sleep-phase duration in percent.

The results are either attached to the primary data or even replace them where appropriated. To avoid data leakages, the *p^2^Health-Cloud* confirms all sent data packages. Occurring uncertainties or missing data sets (caused by e.g. faulty measuring, battery problems or missing sensors) are also handled by the *p^2^Health-Cloud* or the appropriate processing modules.

**Figure 8:**
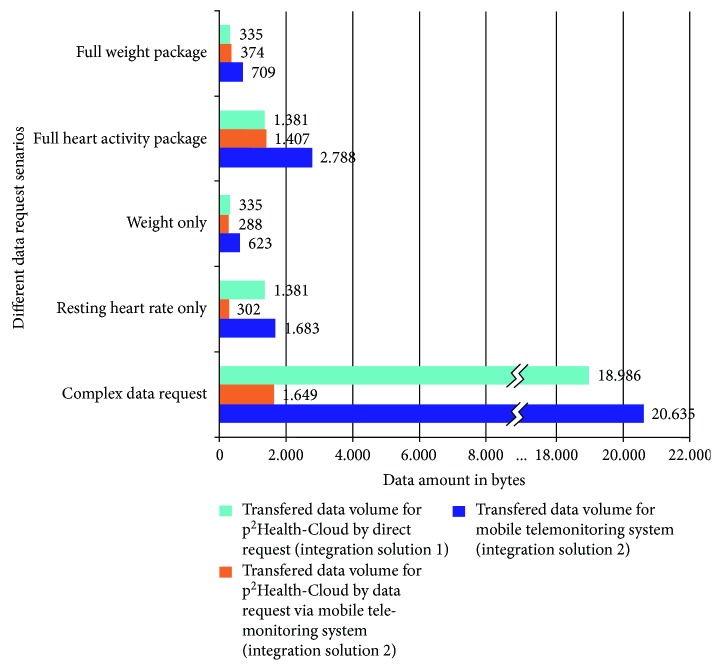
Comparison of the handled data volume depending on selected categories of data sets (package, single value, complex data set) and used integration solutions.

Especially the provider-cloud servers offer data in specific packages, which always include groups of related parameters and additional information (e.g., IDs and pursing data links). Depending on the required data set, a significant data overhead can result, especially if data of different packages are combined.  Figure 8 shows the comparison of the transferred data volume depending on the two introduced integration solutions in Figure 3. For the integration of solution 1, the web-service was treated as part of the *p^2^Health-Cloud* and consequently considered as direct communication between the *p^2^Health-Cloud* and the provider-cloud server. The presented data are generated by data requests with the *Fitbit*-cloud server, whereby initial tests have shown that the similar data concept of *Nokia* leads to similar results. The reported volumes always contain all required request and answer messages for the respective participants.

The chosen data sets can be divided into three categories. In the first category (Figure 8, full packages for weight and heart activity) a complete package request from the *p^2^Health-Cloud* is presumed. In this case, the data selection by the mDCS (solution 2) has almost no effects and the additional communication generates a higher data volume for the *p^2^Health-Cloud* compared to solution 1 due to the higher communication effort between the participants.

In the second category, the focus is only on single parameter requests from the first category packages (Figure 8, weight only and resting heart rate only). Consequently, the data volumes for integration solution 1 are the same as in the first category. For solution 2, the data selection of the mDCS shows a partially significant reduction of the data volume for the *p^2^Health-Cloud* depending on the package size.

The third category shows a complex data-set request in conformity to the planned first real application at the Center for Life Science Automation (University of Rostock). In seven packages, this request includes among others body weight and fat measurements, active and sports phases, resting heart rate, and parameters concerning energy consumption (summary), different heart rate zones, and sleep information. For this specific data-set request, the mDCS reduces the data volume for the *p^2^Health-Cloud* to 9% of the original data volume as in case of integration solution 1. The effectiveness of the data selection depends on the acquired and the required amount of data as well as on the required polling frequency. For most applications, a high reduction of the data volume can be assumed. In any case, the data volume for the mDCS (solution 2) is higher than for the *p^2^Health-Cloud *in solution 1, whereas this is compensated by the fact that every mDCS handles the individual data of one user only.

For the data transfer between the mDCS and the *p^2^Health-Cloud*, the data management reformats the data to an arranged JSON protocol (JavaScript Object Notation), which is already included in the considerations before. JSON offers a simple, compact, and mostly preferred alternative to the tag-oriented XML (Extensible Markup Language) [57]. It is an effective format solution for resources-conserving data transfer [58, 59], which is divided in blocks of single and series data. Every transferred block consists of the following information:

- timestamp of collection,
- source of data (device/provider identification),
- parameter type (as e.g., heart rate, weight),
- measuring unit,
- data value/array of data values.

The data management covers the subjective and objective data entries as well. Therefore, the *MobMedApp* provides questionnaire templates to allow the flexible design of individual questionnaires. Every template includes other input elements as e.g., for binary decisions (yes/no or true/false) or for grading of states (0…100). These templates can be configured by the *p^2^Health-Cloud *for the customer's specific investigations. The configuration includes the questions, their possible answering and execution options (single or multiple execution; if multiple execution then also the repetition rate), the questionnaires' time for prior-notice, and the time-out for overdue questionnaires.

### 3.2. Validation Experiment

In order to validate the presented solution, an experimental application was executed to demonstrate the general feasibility of the presented system and the decentralized approach. The focuses of the examination are hereinafter referred to:

- parallel application of directly and indirectly accessible data sources,
- provision of complementary (for analysis issues) and competitive (for quality-verifying issues) data, which are prepared for further analysis by the *p^2^Health-Cloud*,
- results of the data-selection for this specific application and
- result of the system's battery consumption.

Sleep monitoring is an important subject in preventive medicine [60, 61], especially for investigations of stress and fitness, which often require a resting phase as reference. Consequently, a simplified sleep monitoring is always part of such investigations (baseline) and offers a convenient possibility to validate the confidence and the synchrony of collected data. Hence, a sleep monitoring as example scenario was initiated. The quality of the most commercial wearable sensor devices with sleep tracking function by far still does not reach laboratory standards [62–64] and cannot be used for professional analysis. Anyway, the interest in such compact devices and applications to acquire information about the recovery phases (as e.g., recovery duration and efficiency) is particularly high.

In the example scenario, a wrist-worn *Fitbit Blaze™* (*Fitbit*; San Francisco, USA) with optical heart-rate sensor was applied (indirectly accessible data sources) to acquire heart rate, sleep time, sleep efficiency, and sleep levels (as wake, light, deep, rem). Furthermore, the sensor system *Equivital™ EQ01* (*Hidalgo Ltd.*; Cambridge, UK) was used (directly accessible data sources), which acquires sensor data by a chest belt. Thus, the following specific data are acquired to support the application:

- heart rate, rr-intervals; derived by a two channel ECG via integrated textile electrodes,
- respiration rate; lifting, and lowering of the chest by a strain gauge,
- skin temperature; detected by a thermistor and
- body activity and orientation (as e.g., supine, side and prone posture); determined by an acceleration sensors.

The *EQ01* offers an SDK and an open, proprietary protocol, which allows data reception and an unrestricted configuration of the sensor module. To maintain the full flexibility of the EQ01, the open communication protocol is implemented. Accordingly, also the continuous data transfer of the *EQ01* can be reduced by setting the partial disclosure mode via the mDCS. Thus, all higher resolved signal data as e.g., the ECG, the 3D accelerations, or the strain gauge signal are not sent by the *EQ01*. Even if the sensor module is comparably large and uses *Bluetooth Classic*, the high application potential of the *Equivital™* series regarding parameter provision, system flexibility, and data quality has already been proven in various studies [65–70].

**Figure 9:**
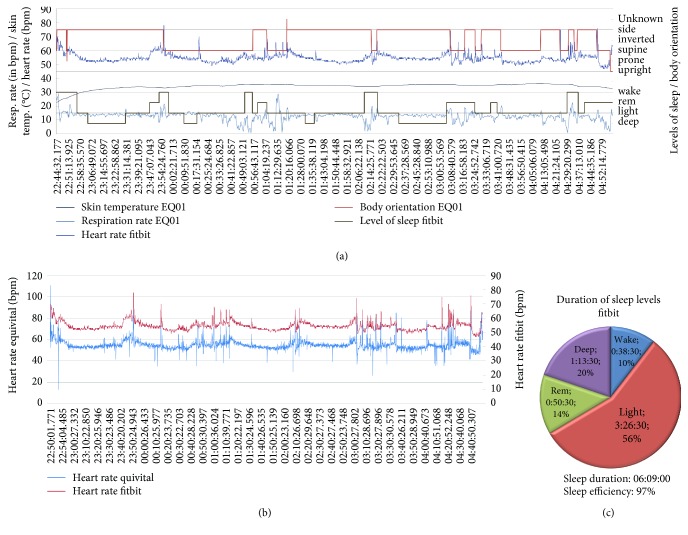
Individual data sets of one subject (1) for overnight monitoring: (a) prepared time-based data for the transfer to the *p^2^Health-Cloud*; (b) comparison of the heart rate from both sensor devices; (c) prepared statistical data for the transfer to the *p^2^Health-Cloud.*

**Figure 10:**
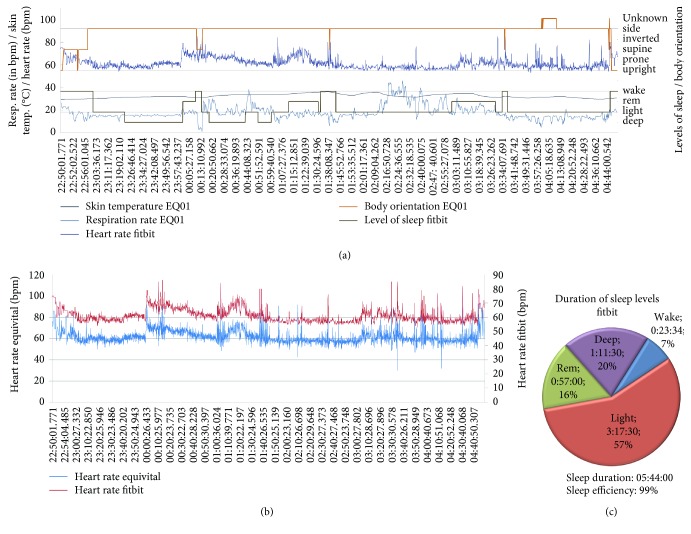
Individual data sets of one subject (2) for overnight monitoring: (a) prepared time-based data for the transfer to the *p^2^Health-Cloud*; (b) comparison of the heart rate from both sensor devices; (c) prepared statistical data for the transfer to the *p^2^Health-Cloud.*

For this example scenario, both sensor systems are worn by four male subjects overnight with the mDCS within range. The *MobMedApp* uses a predefined task table in which the sensor data (as shown in Figures 9 and 10), their resolutions (here: maximum for all parameters), and the upload interval to the *p^2^Health-Cloud* (here: every hour) are configured. This task table and the user-specific data-source table (including the access data for the *Fitbit* cloud server and the *EQ01*) are generated and provided by the *p^2^Health-Cloud,* and are the results of the medics' investigation planning. Further configurations on the *MobMedApp* by users or investigators are not required. Due to the measurement overnight questionnaires and the chronometrage are disabled in the task table for this application.

The selected combination of the chosen sensor devices provides complementary data sets (as e.g., sleep phases, and body orientation), which support the *p^2^Health-Cloud* for the data analysis to detect correlations and to generate intervention measures. In Figures 9 and 10, the preprocessed, individual overnight-data sets of both sensor devices are exemplarily shown for two subjects as they are offered to the *p^2^Health-Cloud*. The presented combination of data sets shows typical reactions for sleep (Figures 9(a) and 10(a)). For example, the low activity level during deep sleep phases can be determined by a consistent body orientation and the low dynamic of both respiration and heart rate. Due to the fact that a chest belt was used for the respiration-rate measurement, the influence of movement artifacts needs to be considered. The *EQ01* permits a more detailed movement analysis by enabling the full disclosure mode and using the 3D acceleration data. The measurement of sleep parameters is provided by *Fitbit* and preprocessed by the *MobMedApps* procedures (percentage of sleep-phase durations and standardization of the designation for body orientation and sleep phases) as shown in Figures 9(c) and 10(c).

Moreover, the system offers the possibility to compare parameters from different sensor devices and measurement methods, respectively, regarding the data quality (competitive data). This is often an important feature for medical staff, especially in medical research, when new sensors devices are introduced and need to be validated. In the example application, the heart rate was parallel acquired on the wrist and on the chest. The data differ concerning provision interval (*Fitbit Blaze™*: ca. one value every sec.; *Equivital™ EQ01*: one value every 5 s, alternatively predictable by rr-intervals) and data resolution (*Fitbit Blaze™*: integer; *Equivital™ EQ01*: one decimal place), but show a high correspondence (Figures 9(b) and 10(b)) in the test application. Furthermore, in all four investigations the heart rate measurement on the wrist shows a lower dependency on movement artifacts (by breathing or usual movements).

#### 3.2.1. Data Usage and Data Selection

Within the presented application experiment the data usage and data selection for both sensor devices are considered in detail for subject 2. The *EQ01* transfers the data in a binary format (payload only) to the mDCS with an average received data amount per hour of 60.5 kB (in partial disclosure mode). This value is influenced by the fitness-level and the activity of the user, which affects the number of occurring rr-intervals and indication values (irregular parameters). However, the data selection takes place before the interpretation of the binary data to avoid unnecessary processing steps. For the above mentioned application, a mean reduction of the transmitted data volume of 80.1% (to 12.0 kB per hour) could be determined. The reason for this high data reduction is that rr-intervals as well as the most indication, status, validity, and administration messages from the sensor are not used in this experiment.

*In case of the Fitbit Blaze*™ only the two *Fitbit* packages for sleep and heart rate are used (see Section 2.3). Thus, a data amount of 47.6 kB per hour (including time stamps) is provided by the *Fitbit* cloud. Depending on the utilization of the data packages, the data amount could be reduced by 35.9%–30.5 kB. The overhead arises from the applied JSON-formatting and unused data as e.g., the detected heart-rate zones and the sleep summary.

Finally, the mDCS transfers the synchronized data (formatted in JSON), including the complementation of time stamps for the *EQ01*-data, the administrative information of the values and the header information, to the *p^2^-Health-Cloud*. The send amount of data per hour for the considered application is 324.8 kB. The high difference of data amount compared to the acquired data is caused by the comprehensive data description to keep the protocol adaptable for different investigation settings and the JSON-format.

#### 3.2.2. Battery Usage

In an additional experiment, the battery usage of the mDCS (smart phone and *MobMedApp*) was examined to estimate the operation lifetime for the executed user application experiment. A *Samsung Galaxy S8* with an octa-core processor (2.3 GHz), 4 GB RAM, a battery capacity of 3000 mAh (claimed talk time: up to 20 h) and the operating system *Android* 8.0.0 (*Oreo*) was chosen. For all experiments, only Bluetooth and Wi-Fi are permanently enabled and used for data transfer (Stamina-mode disabled). During the measurement time the display is turned off. To estimate the app-related battery consumption, the integrated monitoring apps from *Android* are used.

The experiment focuses on the battery consumption of the *MobMedApp* in different configuration cases listed in Table 1. For every configuration case, three eight-hour measurement cycles are executed, in which only the *MobMedApp* and if required the *Fitbit* app are started. Additional tests showed that the *Fitbit* app with less than 1% energy consumption in eight hours does not significantly influence the battery usage. The measurement results shown in Table 1 each also includes the battery consumption for hardware components (as e.g., *Bluetooth*), if applied. These results allow the assessment of the *MobMedApp*'s utility, but it should be noted that they must be considered as an estimation of the battery usage, since the applied battery-monitoring app only offers a limited precision and device-related background processes are not considered in detail or are disabled, respectively. The results were acquired periodically and indicate an almost linear and reproducible behavior.

The comparably high battery consumption of the *MobMedApp* by using the *Equivital™* EQ01 (case 3 and 4) can be traced back to its Bluetooth Classic interface. It is to be expected that the use of BLE devices and a periodical data transmission reduce the battery consumption significantly. However, the executed application setting in combination with a comparable mobile-device configuration assures minimum measurement duration of 24 h without charging.

## 4. Conclusion

This paper deals with the integration and fusion of different data-source interfaces for an IoT-data-science-oriented preventive health-management system. Therefore, the following scientific aspects were considered:

- comparison of structural integration possibilities for indirectly accessible data sources,
- introduction of an approach for the decentralized data source integration and low-level data fusion out of the central processing infrastructure,
- strategies for the management of combined heterogeneous data sources,
- execution of application-relevant evaluation experiments.

A mDCS (smart phone combined with the developed *MobMedApp*) was developed, which realizes data collection in a multi-sensor environment decoupled from the target processing cloud solution (*p^2^Health-Cloud*). The mDCS supports on the one hand the integration of devices with open Bluetooth-interfaces and on the other hand commercial provider-cloud servers (as e.g., *Fitbit* or *Nokia*), which offer interfaces for the collection of individual physical and physiological data from vendor-specific devices. This approach increases the variety of sensor data and reduces the integration effort of the *p^2^Health-Cloud* significantly. In the example solution, it effects a stricter decoupling of the data collection by the mDCS and the data analysis on the cloud-solution.

Therefore, two different concepts regarding the integration of provider-cloud servers and the appropriate data management are discussed in detail. This also comprises the handling of occurring restrictions, additional expenditures (e.g., access limits and complex account handling) and also arising benefits as e.g., the reduction to one interface for several sensors, independent data storage, and time-oriented data requests via the provider-cloud servers.

Beside the function as customer interface (to inform about the customer's status, current result data and necessary preventive intervention measures) the developed *MobMedApp *realizes several data-preparation steps and performs necessary preprocessing routines (as e.g., secondary data generation, data reduction, unit conversion). Thus the further data processing on the *p^2^Health-Cloud* is supported and results in higher system availability. The *MobMedApp *also offers the acquisition of subjective and objective customer's information by investigation-related questionnaires if required.

Initial system experiments show that an individual, decentralized, and parallel data collection from inhomogeneous data sources can certainly be realized by the mDCS. Thus higher data traffic for the user arises and a regular software update for the *MobMedApp* needs to be considered. However, the pursued system structure distributes the individual data management and communication effort out of the cloud solution's scope, supports the system's scalability, and also achieves a higher system's agility regarding upcoming events dependent on the user's individual setting. The individual data amount for the *p^2^Health-Cloud* can be significantly reduced by the *MobMedApp*'sselection function, especially when specific data sets from the provider-cloud servers are requested. Consequently, the usage of the presented solution approach is particularly suitable when several sensor systems (including systems, which allow the data access only via provider-cloud servers) are involved and only small- and medium-sized target-cloud solutions are available.

The presented approach ensures a fast adaptable data acquisition for varying investigation scenarios or conditions for the application site. A higher number of different, integrated devices can further support the system's effectiveness and application potential. First application-related investigations under medical supervision in occupational health with gas and noise-exposed laboratory technicians (considering physical, physiological and environmental data) are planned but still under investigation.

## Figures and Tables

**Table 1:** Battery usage of mDCS (*MobMedApp*) for different configuration cases over eight-hour measurements using the smart phone *Samsung Galaxy S8* (mAh—milli ampere hour).

Configuration case description	Battery usage of *MobMedApp*	Battery usage of mDCS
(1) *MobMedApp* in idle-mode	1.7 ± 0.4% (approx. 51 mAh)	2.8 ± 0.3% (approx. 83 mAh)
(2) *MobMedApp* using *Fitbit*-provider cloud and *p^2^-Health-Cloud*	2.0 ± 0.1% (approx. 60 mAh)	3.8 ± 0.3% (approx. 113 mAh)
(3) *MobMedApp* using *Equivital™* EQ01 (with continuous data transmission) and *p^2^-Health-Cloud*	15.9 ± 0.7% (approx. 477 mAh)	16.7 ± 1.0% (approx. 500 mAh)
(4) *MobMedApp* using *Fitbit*-provider cloud, *Equivital™* EQ01 (with continuous data transmission) and *p^2-^Health-Cloud*	18.9 ± 0.7% (approx. 565 mAh)	19.8 ± 0.9% (approx. 595 mAh)

## Data Availability

The data used to support the findings of this paper are available from the corresponding author upon request.
